# Anthropogenic Habitat Disturbance and Ecological Divergence between Incipient Species of the Malaria Mosquito *Anopheles gambiae*

**DOI:** 10.1371/journal.pone.0039453

**Published:** 2012-06-22

**Authors:** Colince Kamdem, Billy Tene Fossog, Frédéric Simard, Joachim Etouna, Cyrille Ndo, Pierre Kengne, Philippe Boussès, François-Xavier Etoa, Parfait Awono-Ambene, Didier Fontenille, Christophe Antonio-Nkondjio, Nora J. Besansky, Carlo Costantini

**Affiliations:** 1 UMR MIVEGEC (UM1, UM2, CNRS 5290, IRD 224), Institut de Recherche pour le Développement (IRD), Montpellier, France; 2 Laboratoire de Recherche sur le Paludisme, Organisation de Coordination pour la lutte contre les Endémies en Afrique Centrale (OCEAC), Yaounde, Cameroon; 3 Department of Biochemistry, University of Yaounde I, Yaounde, Cameroon; 4 Department of Geographical Research, Institut National de Cartographie (INC), Yaounde, Cameroon; 5 Eck Institute for Global Health, Department of Biological Sciences, University of Notre Dame, Notre Dame, Indiana, United States of America; Instituto de Higiene e Medicina Tropical, Portugal

## Abstract

**Background:**

Anthropogenic habitat disturbance is a prime cause in the current trend of the Earth’s reduction in biodiversity. Here we show that the human footprint on the Central African rainforest, which is resulting in deforestation and growth of densely populated urban agglomerates, is associated to ecological divergence and cryptic speciation leading to adaptive radiation within the major malaria mosquito *Anopheles gambiae*.

**Methodology/Principal Findings:**

In southern Cameroon, the frequency of two molecular forms–M and S–among which reproductive isolation is strong but still incomplete, was correlated to an index of urbanisation extracted from remotely sensed data, expressed as the proportion of built-up surface in each sampling unit. The two forms markedly segregated along an urbanisation gradient forming a bimodal cline of ∼6-km width: the S form was exclusive to the rural habitat, whereas only the M form was present in the core of densely urbanised settings, co-occurring at times in the same polluted larval habitats of the southern house mosquito *Culex quinquefasciatus*–a species association that was not historically recorded before.

**Conclusions/Significance:**

Our results indicate that when humans create novel habitats and ecological heterogeneities, they can provide evolutionary opportunities for rapid adaptive niche shifts associated with lineage divergence, whose consequences upon malaria transmission might be significant.

## Introduction

Environmental change is a driving force in speciation and the genesis of evolutionary novelty, prompting adaptive variation to emerge by means of natural selection [1]. Today humans represent one of the Earth’s most important disruptive forces upon the natural environment [2], impacting the evolutionary trajectories, and accelerating the genetic and phenotypic divergence of many organisms [3]–[5]. It is largely accepted that human-induced environmental changes and habitat destruction can cause species extinction and alter the structure of communities [6]. However, even in well-studied examples like tropical forest disturbance and clearance, the impact on biodiversity is not fully understood [7]. Colonisation of new environments–directly or indirectly mediated by human action–can facilitate ecological diversification and rapid speciation as a by-product of adaptation to divergent selective regimes [8]. Anthropogenic modifications of the environment can lead to ecological divergence and ultimately foster the rapid evolution of reproductive isolation between ecotypes [9]. However, this process is difficult to document in nature unless it is caught in action [10]. Here we have used ongoing cryptic speciation in forest populations of the most important afro-tropical malaria mosquito *Anopheles gambiae sensu stricto* to demonstrate that recent landscape transformations in the African equatorial rainforest, due to the growth of densely populated urban areas, are at the heart of adaptive ecological divergence of two incipient sibling species within this taxon.

Genetic subdivisions marking incipient speciation within *An.* *gambiae* have long been recognised [11], [12]. The rarity of ‘hybrids’ and heterogamous matings [13] in natural field populations led to the recognition of two isomorphic ‘molecular forms’, named M and S, representing diverging evolutionarily significant reproductive units within this mosquito [14]. The two forms are recognised based on fixed differences in the IGS sequence of rDNA [15], [16]. In most of their distribution range, M and S are sympatric [17]. Genetic and ecological evidence suggest that divergence in M and S occurs in the face of the homogenising action of gene flow, through the selection of genes conferring ecological adaptation and controlling reproductive isolation [18]–[21]. However, an alternative model posits that complete absence of effective ongoing gene flow is compatible with the patterns of heightened genetic differentiation between M and S observed in three unlinked portions of the genome characterised by reduced recombination (i.e. the pericentromeric ‘speciation islands’ observed in all three chromosomes of *An. gambiae*) [20], [22], [23]. In order to gain a better understanding of the processes underlying M and S genetic differentiation, it is necessary to investigate the ecological causes and consequences of their divergence.

In the West African savanna, ecological divergence of M and S has been associated to eco-geographical clines. The M form dominates in the most xeric habitats, whereas the S form is more abundant in less arid regions [24]. In the savanna, ecological divergence between M and S is also expressed at a different geographical scale. While the S form breeds mainly in small, ephemeral, rain-dependent puddles, the M form predominates in areas characterised by larger, more temporally stable breeding sites mainly of anthropogenic origin, such as rice paddies and other water reservoirs related to irrigation [25], [26]. These physiognomic differences among larval habitats are associated to differences in the composition and abundance of mosquito predators [27], which are believed to drive the divergent selection response of natural populations of M and S responsible of their ecological segregation [27], [28].

Populations of M and S in the rainforest both correspond to a chromosomal inversion cytotype distinct from savanna populations, characterised by the homosequential standard arrangement [12]. In puzzling contrast to savanna populations, no apparent habitat segregation has been identified in forest populations [29], despite strong evidence for genetic differentiation and reproductive isolation [18], [29], [30]. However, ecological niche modelling identified variables related to human activity, such as distance to populated places or roads, as the best predictors of *An. gambiae* occurrence [24], [29], suggesting that environmental heterogeneity associated with human-induced habitat disturbance could identify candidate ecological gradients segregating M from S in the rainforest. Because deforestation and urbanisation are major processes that are profoundly modifying the equatorial rainforest [31], [32], we predicted that M and S might be diverging according to such ecological gradients.

In what follows we report that anthropogenic changes of the central African rainforest are associated to ecological divergence between M and S. We have correlated the probability of occurrence of the two molecular forms to an index quantifying the amount of urban land cover to demonstrate that M and S segregate along an urbanisation gradient forming a bimodal cline. Urbanisation is generally defined as the physical growth of urban areas as a result of environmental change and the concentration of the human population in towns. Here, however, we consider urbanisation as a change in landscape patterns across space rather than its temporal dynamics, although urban habitats are of course the outcome of modifications of the original natural landscape through time. In our context, urban areas result from several concomitant processes including deforestation, building-up of infrastructures, and increase of human population density. These processes leave identifiable signatures upon the natural landscape that can be extracted from remotely sensed data, which we have exploited to characterise the environmental gradients along which the two molecular forms segregate. In this work, we show also that the M form is adapting to polluted larval habitats present in the most densely urbanised settings from which it has not been historically recorded, indicating that lineage divergence in this mosquito is associated to rapid adaptive niche shifts induced by anthropogenic habitats.

## Results

### Ecological Divergence of the Molecular Forms

**Figure 1:**
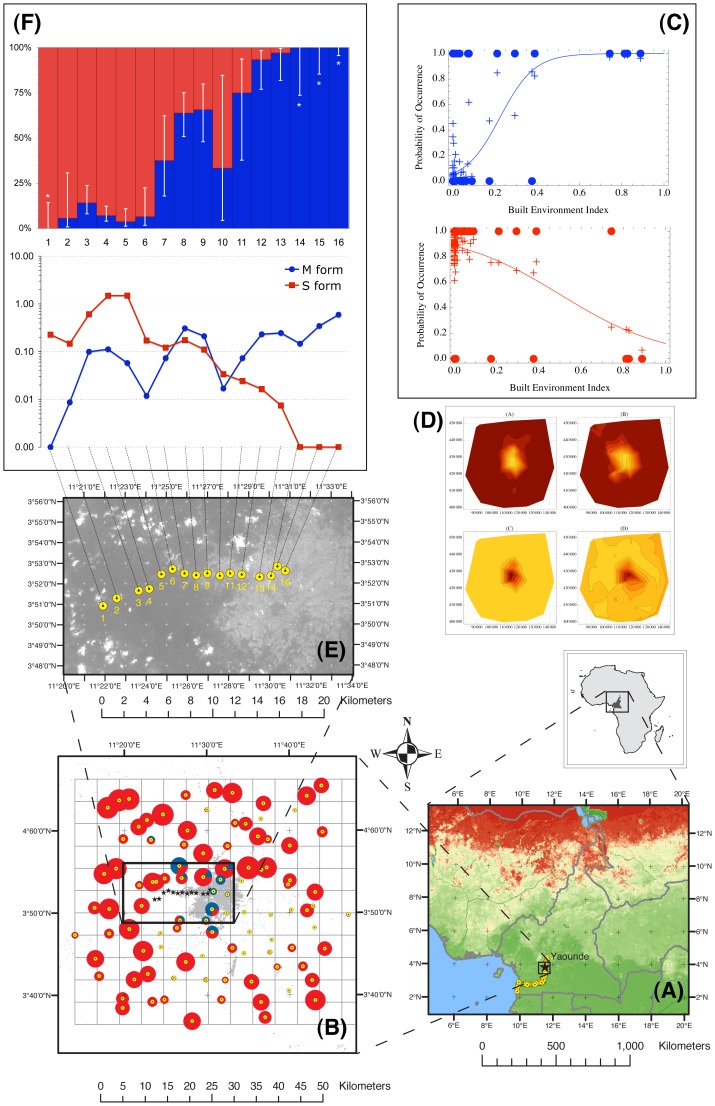
Distribution and abundance of the molecular forms of *Anopheles gambiae* in the rainforest of Cameroon, investigated at different geographical scales. (A) location of the meso-geographic survey area (square), and the sampled sites (dots) serving as the evaluation data set for model validation; dark green individuates the highest values of percentage tree cover outlining the limits of the forest domain (source: Global Land Cover Facility www.landcover.org); (B) relative abundance of M (blue) and S (red) in the meso-geographic survey area. The built environment land class extracted from satellite images is depicted as grey pixels. Dots are sampled localities; those without associated pies returned no *An. gambiae*. The size of the pies is proportional to the total number of *An. gambiae* specimens that were collected in each location. The insert shows the localities (stars) and limits of the micro-geographic survey; (C) binary logistic models fitted to the M (blue) and S (red) occurrence data of Figure 1B, in relation to the proportion of surface occupied by the built environment land class. Crosses denote fitted probabilities of an autologistic model taking into account spatial autocorrelation in occurrence; (D) contour plots showing the distribution of interpolated probabilities of occurrence of M (above) and S (below) in the survey area of Fig. 1B. Bright yellow corresponds to highest, and dark red to lowest probability of occurrence. Plots on the left refer to the ordinary logistic (continuous lines in Fig. 1C), those on the right to the autologistic (crosses in Fig. 1C) models; (E) location of 16 sampled localities along a rural to urban transect, superimposed on a SPOT-5 satellite image; (F) relative proportion (±95% confidence limits) and relative density, expressed as mean number of mosquitoes per sampled house, of adult M (blue) and S (red) along the micro-geographic rural to urban transect.

To test the hypothesis that forest populations of the molecular forms of *An. gambiae* segregate along anthropogenic environmental gradients, we surveyed a 50×50 km area around the capital city of Cameroon, Yaounde (Fig. 1A), a region that was previously identified to be equally suitable to both M and S [29]. One hundred localities scattered on a lattice with Yaounde at its centre (Fig. 1B) were sampled to study spatial patterns of occurrence of the two molecular forms. A negative species association coefficient (Hurlbert C_8_ = –0.412; *P* = 0.04 by Fisher’s exact test), indicated that the two forms co-occurred in the same locations less than expected from a random distribution across locations, given their marginal frequencies in a 2×2 contingency table summarising the forms presence/absence in each locality [33]. Moreover, species occurrence was spatially structured, as the frequency of each form in a locality was not independent of its frequency at surrounding localities (Moran’s *I* [34] test for spatial autocorrelation: M, *I* = 0.276, *P*<0.001; S, *I* = 0.242, *P*<0.001). The M form (*n* = 35) clustered in 11 (15%) contiguous localities in the urbanised settings, while the S form (*n* = 707) occurred in 93% of the samples, but it was completely absent from the core of Yaounde. Co-occurrence of the two forms was limited to ten localities in the peri-urban area (Fig. 1B). The shape of the resulting spatial correlograms (Fig. S1) expressed by Moran’s *I* statistic were indicative of patchiness, and compatible with a large patch of ∼15–20 km size [35]. A Landsat 7 ETM+ satellite image was used to extract a land use class defined as “built environment”. This class encompasses human infrastructures, including buildings, open spaces, and transportation features. The proportion of landscape classified as “built environment” in each spatial unit served as an explanatory variable (“Built Environment Index”–BEI) to measure the shape and strength of association between the degree of urbanisation and the probability of occurrence of M and S. A binary logistic model fitted to the data demonstrated that the probability of occurrence of the M form was positively correlated with the BEI, and–conversely–the probability of occurrence of the S form was negatively correlated with the BEI (Fig. 1C). In the study area, therefore, M and S segregated along an ecological gradient correlated with the degree of urbanisation (Fig. 1D).

To investigate the stability of ecological segregation at a finer degree of spatial and temporal resolution, we studied the dynamics of larval and adult *An. gambiae* molecular forms along a 18-km transect ranging from the core of the urban habitat to the nearest rural locales in western Yaounde (Fig. 1E). Sixteen sites, approx. equally spaced along the transect, were surveyed monthly across a whole year. A SPOT-5 satellite image was used to calculate the BEI in 1×1-km quadrats centred on the sampled sites. The analysis of 723 adult and 3,282 immature *An. gambiae* confirmed the divergence of the molecular forms along the built environment gradient. The segregation pattern of larvae and adults mirrored each other almost perfectly (Fig. 1F and Fig. S2): the M *vs*. S relative frequency changed gradually along the transect forming a bimodal cline. The S form was exclusive to the rural habitat, whereas only the M form was present in the core of the densely urbanised settings of central Yaounde. No adult and only four larval M/S hybrids were recorded in the peri-urban zone of sympatry. The centre of the cline was identified in the peri-urban area between localities 7 and 8 of Fig. 1E, and its width was estimated to span ∼6.1 km.

### Temporal Dynamics of Ecological Divergence

**Figure 2:**
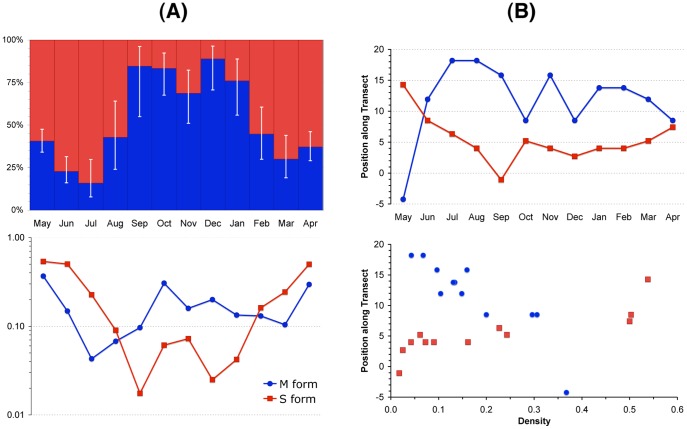
Temporal distribution and abundance of the molecular forms of the malaria mosquito *Anopheles gambiae* in the rainforest of Cameroon, investigated across one year. (**A**) Relative abundance of M (blue) and S (red) by month of collection, starting on May, 2008. The relative proportion (±95% confidence limits) is shown above, the relative density, expressed as mean number of mosquitoes per sampled house, is shown below; (**B**) position along the rural to urban transect (in arbitrary units corresponding to the identification codes of locations in Fig. 1E) of the median probability of occurrence of M (blue) and S (red) by month of collection (above), and mean *An. gambiae* density (below).

**Figure 3:**
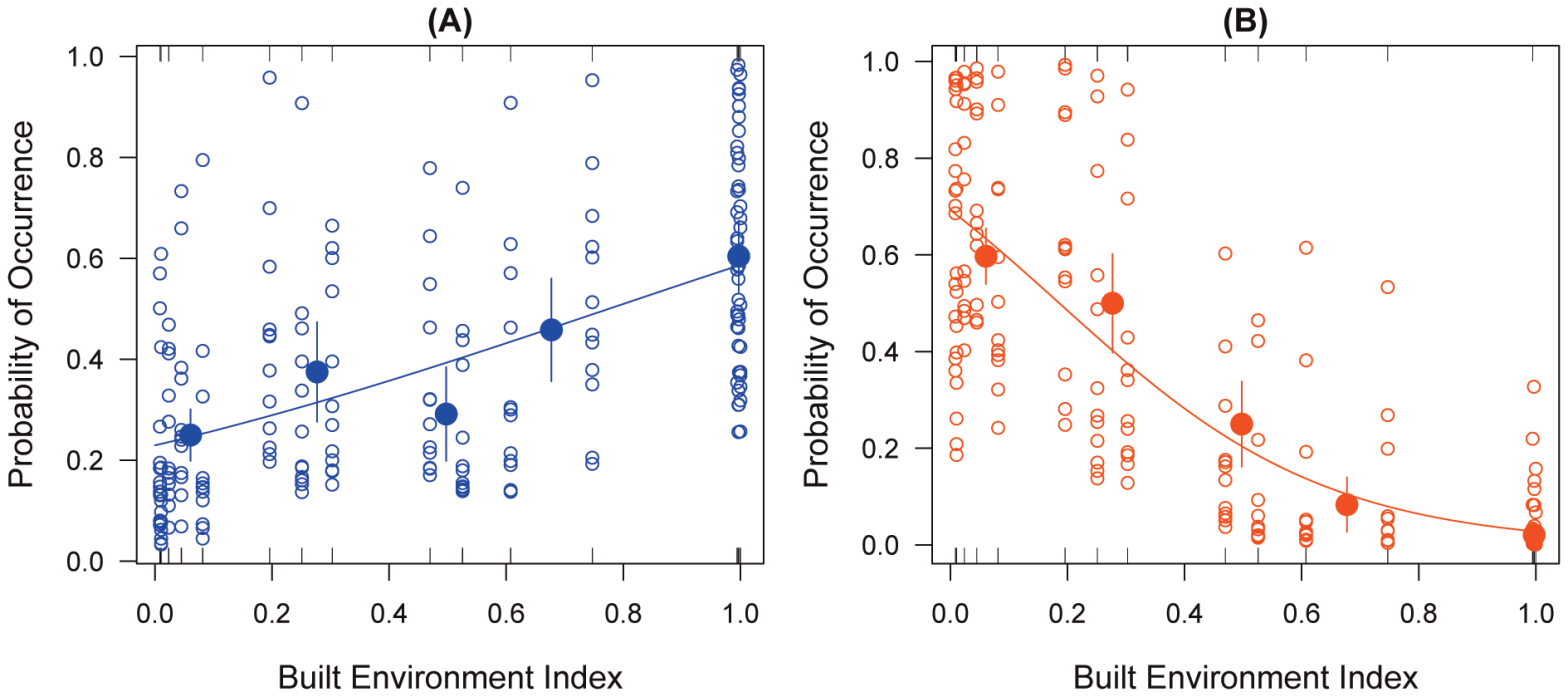
Binary logistic regression models showing the estimated probability of occurrence of (A) adults of the M form (blue open dots, closed circles and continuous line); and (B) adults of the S form (red open dots, closed circles and continuous line) in relation to the Built Environment Index (BEI) calculated for 1×1 km quadrats including the 16 sites of the micro-geographic rural to urban transect of Fig. 1E. Tick marks on the floor and ceiling of each scattergram visualise occurrences (0 = absence, 1 = presence); larger thickness of the tick marks denotes higher frequency. Open dots represent the estimated probability of occurrence of the minimal adequate models including the BEI, *Anopheles gambiae* population density, and sampling effort as explanatory variables. The regression lines visualise the fitted probability of occurrence when only the BEI is included as explanatory variable. Closed circles show the mean observed response of occurrence (± standard errors) for five equally spaced classes of the BEI for visual assessment of ‘goodness-of-fit’.

Despite large asynchronous fluctuations in each form abundance (Fig. 2A), spatial segregation persisted throughout the survey. Binary logistic regression confirmed the results of the meso-geographic survey for the association between the probability of occurrence of the two forms and the built environment index (Fig. 3). Despite large variations in the fitted probability of occurrence due to fluctuations in the forms’ detectability, as expressed by the average mosquito density and sampling effort deployed, the BEI contributed to a significant proportion of the explained deviance (Table S1) in both M (31%) and S (67%).

The S form exhibited greater fluctuations in abundance compared to the M form (Fisher F-test, F-ratio = 3.8; 95% CI: [1.1, 13.3]; *P* = 0.035–Fig. 2A). By modelling the probability of occurrence of each form along the transect in relation to the month of survey, we explored the extent and nature of the temporal changes in spatial segregation between the forms. We estimated from each binary logistic model the position along the transect corresponding to the median probability of occurrence of each form, and plotted this endpoint in relation to the month of survey, or M and S abundance (Fig. 2B). We used this endpoint as a descriptor of the ‘front’ of the limits of each form distribution along the transect. The position of the median probability of occurrence along the transect changed across time more in the case of M than S (Fig. 2B), but the difference was not statistically significant (standard deviation *s* = 6.1 for M, and *s* = 3.7 for S; Fisher F-test for equality of variances F_11,11_ = 2.72; *P* = 0.11). However, the position of the boundary was strongly correlated with M and S density (Pearson product-moment correlation coefficient *r* = –0.890; 95% CI: [–0.969, –0.646] *P*<0.001 for M; and *r* = 0.845; 95% CI: [0.526, 0.955]; *P*<0.001 for S–Fig. 2B). These results indicate that when population abundance was less, the two forms retracted in their respective core habitat, and, conversely, when densities were higher, they expanded towards less suitable habitat.

### Validation of the Form-habitat Relationship Based on the Built Environment Index

To test the predictive properties of the binary logistic models parameterized on the Yaounde transect data set (Table S2) when extrapolated to a larger spatial extent, we calculated from the models the expected probability of occurrence of the two forms in 200 localities covering the forest domain of southern Cameroon (Fig. 1A), and compared them to actual occurrences observed during previous surveys carried out in October-December 2005 [29] and 2006. The built environment index was extracted from a mosaic of three Landsat ETM+ images on which a grid of 5×5 km cells was superimposed. The model with the BEI included as one of the predictors could correctly discriminate between occupied and unoccupied sites 73% and 68% of the time for M and S, respectively, with both values being significantly greater at *P*<0.05 than the 50% random expectation. This was indicated by the values of the Area Under the Curve (AUC) of the Receiver Operating Characteristic (ROC) graphs: for M, AUC = 0.727 (95% confidence interval: [0.630, 0.824]), and for S, AUC = 0.679, [0.605, 0.753] (Fig. S3). Removal of the BEI from the model caused a reduction in its predictive performance, significantly so in the case of S (AUC = 0.701 [0.602, 0.801] for M, *Z* = 0.86, *P* = 0.19 one-tailed; and AUC = 0.603 [0.523, 0.683] for S, *Z* = 2.40, *P* = 0.01 one-tailed). More details about model validation are included as Supporting Information in Text S1. These results confirm and generalise the conclusions of the meso- and micro-geographic surveys around Yaounde, which found that the density of built environment is a significant explanatory variable for the observed pattern of spatial segregation of M and S. The greater impact of the BEI on model performance for S, and the asymmetry between forms in the probability of occurrence in relation to the BEI suggest that factors associated with the urban environment limit colonisation of this habitat in the case of S, whereas M behaves more opportunistically in response to prevailing environmental conditions.

### Ecological Niche Expansion of the M Form

The spatial and temporal stability of divergence, the similarity of segregation patterns of adults and immatures, and the narrowness of the contact zone relative to the potential dispersal abilities of *An. gambiae* [36], indicate a process of environment-dependent selection and local adaptation maintained by physiological, behavioural, or other life-history traits conveying a fitness advantage in each alternative habitat. Presently, we do not know what are the precise mechanisms underlying this pattern. We have some evidence, however, that M has evolved greater tolerance to environmental stressors occurring in the urban habitat.

**Figure 4:**
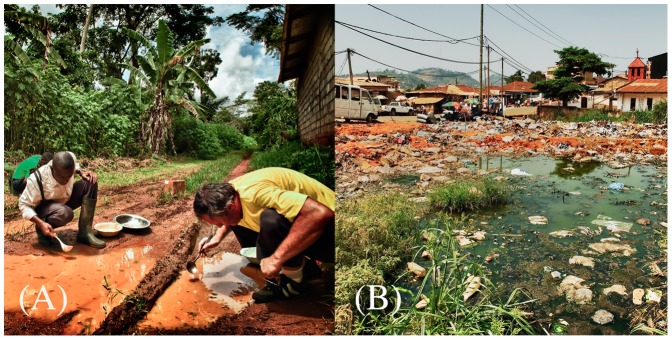
Larval habitats of the malaria mosquito *Anopheles gambiae* in the rainforest of Cameroon. **(A)** field entomologists ‘dipping’ larvae from the typical rural rain-dependent puddles and ruts where the S form breeds. **(B)** water collection in an urban dumping ground where the M form breeds. The two sites in the picture are situated respectively in location No. 1 and No. 14 of Fig. 1E. The subjects appearing in panel (A) are among the authors of the paper (BTF and PB) and have given written informed consent (as outlined in the PLoS consent form) to publication of their image.

**Figure 5:**
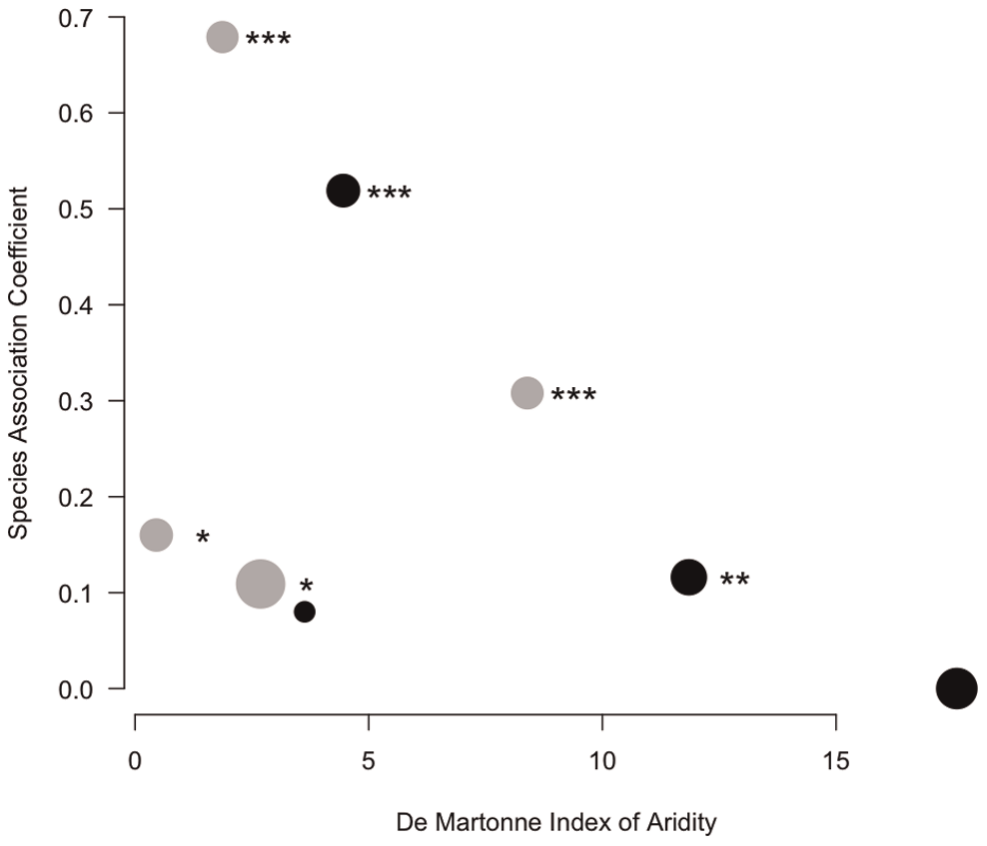
Species association analysis. Hurlbert coefficient of species association C_8_, calculated from presence/absence of *Anopheles gambiae* and *Culex* spp. (mostly *Cx quinquefasciatus*) in urban larval habitats, plotted against an index of aridity calculated on a per-month basis. Lower values of the index indicate greater aridity. The size of the points in the plot is proportional to sample size (i.e. number *n* of sampled breeding sites; smallest point: *n* = 76; largest point: *n* = 173). The colour of the points indicates the town where the samples were collected (black = Douala, grey = Yaounde). Values of C_8_ significantly greater than statistical independence of occurrence (C_8_ = 0) are marked with asterisks. *P<0.05; **P<0.01; ***P<0.001.

Larvae of *An. gambiae* typically develop in small, ephemeral, unpolluted sunlit puddles formed by the accumulation of rain water on open ground (Fig. 4A). Unexpectedly, in urban settings we found larvae of the M form successfully developing in waste waters typically derived from polluting human activities (Fig. 4B). There is hardly any historical record of *An. gambiae* occurring in larval habitats contaminated with large amounts of decaying organic matter in Cameroon or Central Africa [37], [38]. Ecologists use species associations as a framework to synthesise and predict environmental characteristics. Associations may be good predictors of environmental conditions, even in the absence of any direct biological interaction between individual species [39]. In this context, the urban mosquito fauna in tropical Africa is dominated by *Culex quinquefasciatus*, a pest whose larvae commonly develop in cesspits, sewage drains, or waste-water pools, where decaying organic matter is plentiful. Several other mosquitoes of the genus *Culex* (*duttoni*, *nebulosus*, *tigripes*) can be found associated to this species in larval habitats contaminated with decaying organic matter, and it is reported that *An. gambiae* larvae are replaced by *Culex* as soon as organic pollution exceeds a certain threshold [37], [40]. To assess current levels of habitat similarity between *Culex* and *An. gambiae* immatures in urban areas, we estimated their degree of association during parallel larval surveys carried out in both major towns of Cameroon, Douala and Yaounde. We observed some variability in the degree of association, nevertheless six out of eight species association coefficients were significantly greater than zero, indicating that *Culex* and *An. gambiae* co-occurred in the same breeding sites more than expected from chance alone (Fig. 5). The value of the index of association was somewhat correlated with season. We postulated that under conditions favourable to the availability of suitable larval habitats, *An. gambiae* M may have a propensity to avoid environmental stressors and competition from *Culex* by breeding proportionally more in less polluted habitats. Whenever suitable larval habitats become scarce, however, *An. gambiae* M can occupy also more polluted breeding sites, thereby increasing its strength of association with *Culex*. Suitable larval habitats for *An. gambiae* are less abundant during dry periods characterised by less rain and increased evaporation. Thus, we measured the strength of the correlation between an index of aridity, which was used as a proxy for availability of temporary water collections, and the species association coefficient (Fig. 5). In agreement with our working hypothesis, we found a pattern for increased strength of association between *Culex* and *An. gambiae* with greater aridity, however we could not reject the null hypothesis of no correlation at *P*<0.05 (Spearman rank correlation coefficient *r*_s_ = –0.548; *P* = 0.17). It is interesting to note that larval surveys carried out in Yaounde between 1948 and 1952 estimated the odds of recording an *An. gambiae* breeding site compared to a *Culex*-only breeding site at 1∶6 [40], whereas in our survey the odds rose to 3.5∶1 in Yaounde and 6.3∶1 in Douala. Overall, current evidence indicates that in the forest of Cameroon the urban form M of *An. gambiae* is presently found to significantly co-occur at times with *Culex* mosquitoes adapted to highly polluted larval habitats, with whom it was not historically recorded to share the same kind of breeding sites.

## Discussion

### Ecological Divergence and Adaptive Radiation in *An. gambiae*

Much of our current understanding of the *An. gambiae* M and S forms is based on populations inhabiting the dry savannas of West Africa. There, ecological divergence between forms appears driven by adaptation to alternative larval habitats that differ in their stability and complexity: small, ephemeral, rain-dependent puddles barren of predators in the case of the S form, and larger, longer-lasting, anthropogenic water bodies richer in predators in the case of the M form [25], [26], [28]. These differences are embedded in the molecular forms’ divergent geographical distribution along aridity clines [24]. Genetic distinctiveness of forest relative to savanna populations of M and S is reflected in the different degree and nature of chromosomal polymorphism [12], [24], [29], as well as in significant DNA-level differentiation between forest and savanna M populations [41]–[43]. Moreover, present knowledge about the geographical range of forest and savanna M populations points to their allopatry or at most parapatry across central and western Africa [17], and certainly so in Cameroon [29]. This indicates that forest and savanna M populations are currently evolving along independent trajectories. However, although M and S are at least as distinct genetically in the rainforest domain [18], [30], [41], until now ecological variables associated to such differentiation had not been clearly established [29].

Our results demonstrate strong ecological divergence between forest populations of the M and S molecular forms that likely resulted from niche expansion of the M form into the urban environment. This process must be very recent as profound changes of primeval landscapes in the African rainforest have occurred only during the last century. The exponential demographic explosion of urban centres is consequent to high population growth rates and spontaneous immigration of rural populations in search of better living conditions during the period spanning the end of World War II and independence [44]. Historical records also indicate that niche expansion has been associated to adaptations allowing M to develop in urban larval habitats that were not previously occupied by *An. gambiae*. The polluted nature of these novel habitats suggests that adaptive changes involve probably the evolution of greater tolerance to environmental stressors, and, perhaps, better ability to compete successfully with mosquitoes that are well adapted to live in such polluted habitats.

These observations suggest that the proximate ecological factors underlying M and S divergence are likely to be different in forest as compared to savanna populations. Disruptive selection for life-history traits and behavioural mechanisms diminishing the risk of predation in more complex and stable larval habitats has been proposed to operate in *An. gambiae* populations inhabiting the arid savanna of West Africa [27], [28]. We speculate that this is probably not the case for populations occurring in the humid forest of southern Cameroon. We have preliminary evidence, in fact, that in Yaounde M and S respond differently to environmental stressors occurring in urban larval habitats, with M exhibiting–according to expectations–higher tolerance than S to pollutants derived from decaying organic matter (B. Tene-Fossog, C. Antonio-Nkondjio, P. Kengne, F. Njiokou, N.J. Besansky, C. Costantini, unpublished data). Moreover, we have also preliminary evidence that in our study area top predators are often absent from *An. gambiae* breeding sites [45], regardless of the degree of urbanisation of the area in which they occur (B. Tene-Fossog, C. Antonio-Nkondjio, P. Bousses, N.J. Besansky, C. Costantini, unpublished data). Nevertheless, it is still premature at present to conclude that similar ecological processes and selective forces are not in place in both eco-geographical domains until results from further studies addressing this question will become available.

At the demographic level, our results show that the distribution of M in the forest can be described as a mosaic of populations forming a large meta-population in urban ‘ecological islands’, whereas the S form constitutes a network of populations in the surrounding rural villages within the forest ‘ocean’. A narrow contact zone of strict sympatry occurs at the ecotone between urban areas and the forest. The contact zone is larger at times of demographic increase, and gets narrower (or it may even disappear) as populations wane off. These spatial and demographic attributes can explain why genetic differentiation between populations in the forest is greater in M than S [30], as well as the large variance in relative frequency between locales that has been frequently reported for M and S forest populations [17]. Moreover, they reveal that levels of sympatry between M and S populations can be dynamic, suggesting that the degree of hybridization between M and S may fluctuate following the extent of contact between these populations.

### Conclusions and Perspectives

The main aim of this study was to identify major environmental gradients of ecological segregation between cryptically speciating populations of *An. gambiae*, rather than to build accurate distribution maps of M and S (see Supporting Information Text S2 for a discussion about the methodological limitations of this study). In this respect, the density of built environment has proved to be a good descriptor of M and S ecological divergence in the rainforest domain of Cameroon. Better environmental predictors of this pattern and the functional mechanisms explaining this process await discovery. The significance of this process lies in the fact that human disturbance of the original forest environment is resulting in novel opportunities for adaptation associated to ecological and genetic diversification of this biological model of utmost importance to human health.

The potential epidemiological consequences of this process should not be ignored. Malaria transmission in modern urban centres in Africa is much less intense than in rural areas, corresponding to significant differences in malaria morbidity and mortality between rural and urban populations [46], [47]. However, peculiar breeding sites of *An. gambiae* have been recently identified as novel adaptations to the urban habitat [48], [49]. Because the close interaction with humans has been recognised as a major evolutionary force shaping the evolution of *An. gambiae* [50], adaptation of this mosquito to urban environments constitutes a threat that might seriously impact human health. The relationship between vector population parameters and malaria epidemiological endpoints like transmission, morbidity, or mortality is very complex and not fully discerned [51]–[53]. Yet, the widespread occurrence of historically unrecognised sources of larval development in the urban environment, accompanied by the global trend leading to the demographic and spatial growth of densely populated urban centres, suggest that a paradigm shift in malaria transmission and control in Africa may occur in the future: from a disease affecting mainly rural settings of low host density to densely populated urban areas where humans and mosquito behaviours are likely to create new opportunities for parasite transmission.

## Materials and Methods

### Meso-geographic Survey

In order to investigate the spatial structure of occurrence of the two molecular forms in a region surrounding the capital of Cameroon, Yaounde, a stratified randomised sampling plan was conceived and implemented in the field by a three-stage approach. During the first stage, the study area was defined and partitioned in 100 isometric cells of 25 km^2^ by a 5×5-km grid which served as a sampling frame. The size of the cells was chosen based on prior information on *An. gambiae* dispersal [36], with the objective to define independent sampling units. The frame embodied the regular component of the sampling plan, whose aim was to stratify the geographical space in order to achieve nearly homogenous coverage by systematic sampling over the study area.

Next, the localities to survey were drawn at random from the list of all populated places contained in each cell with the aid of ancillary data and a Geographical Information System (GIS). The objective of this stage was to reduce sampling bias associated with the specific choice of the localities contained within each cell of the grid. We aimed to select only one locality per cell based on additional criteria like threshold human population size (>100 inhabitants), and accessibility.

The third stage was the actual field survey, when geographical information was validated and updated to correct discrepancies, or to add new data. Field data were then entered in an ‘output’ GIS database exploited for statistical analyses. Details of how each stage was implemented are further described as follows.

In order to conceive the sampling plan and to assist the field survey, several layers of geographic information, projected in Universal Transverse Mercator (UTM) zone 32N, were entered and managed in ArcGIS (ESRI; http://www.esri.com) to build a map of the study area. A Landsat 7 ETM+ image (1∶60,000) taken on May 18, 2000 was used as a background layer over which the transportation and hydrographic networks were generated, respectively, from road maps (1∶200,000), and remote sensing data downloaded on May 2003 from the Shuttle Radar Topography Mission website (http://www2.jpl.nasa.gov/srtm/). The layer of populated places was generated from topographic maps created between 1970 and 1980, data available at the National Institute of Cartography of Cameroon (*Institut National de Cartographie*–INC), and the GEOnet Names Server gazetteer as detailed below. These data were subsequently validated by ground-truthing during the field surveys.

To achieve a random selection of the localities to be surveyed in each cell of the sampling grid, we used the georeferenced databases of populated places available at INC and from the GEOnet Names Server (http://earth-info.nga.mil/gns/html). “Populated places” (hereafter also “localities”) correspond to geographical elements (point features in the ancillary databases) with associated toponyms that spatially characterise human settlements. In rural settings, these geographical elements correspond to what would generally be defined as “villages”, small settlements that in our study area rarely extend over more than one square kilometre, and usually much less (cf. built environment map in Fig. 1B). In urbanised settings, however, these features represent city quarters or neighbourhoods. One toponym was selected at random in each cell from the list of localities listed in the ancillary database.

Field collectors visited the selected localities following their toponym and ancillary geographical coordinates; they collected indoor-resting mosquitoes in a few patches of human dwellings located in the vicinity of potential mosquito larval habitats and within a radius of *c.* ±100 m from each other. The geographical coordinates of a locality were taken with a GPS receiver at the approximate centroid of the patch of houses from where collections took place. The corresponding geographical and entomological data were then aggregated in the output GIS database as a point feature to represent that locality.

Due to deficiencies in the geographical data available for this region of the world, not all the localities that were selected *in silico* could be effectively sampled in the field. On a few occasions, one of the selected localities was replaced by the nearest neighbour because it was missing or inaccessible. Sometimes ancillary geographical coordinates were not accurate; in this case, the selected locality was looked for in the field by matching toponym. These constraints explain why in Fig. 1B a few of the cells of the sampling grid contain more than one sampled locality, or some cells do not contain any locality. A few of the localities validated on the ground fell out of the original 10×10 sampling frame; accordingly, this was extended by appending rows or columns of adjoining cells, thereby forming a new 11×12 sampling frame (Fig. 1B). The final effective average median distance of each sampled locality from its neighbours was 6.3 km (IQR: 5.5–7.2 km), which is close to the 5 km target defined at the outset with the choice of a 5×5 km cell size.

Female resting mosquitoes were collected inside human dwellings from late May to mid July 2007 by insecticide space sprays [54]. On average, four houses (range: 1–8) and 2–18 sleeping rooms from two to three locations were sampled daily; sampling effort was increased whenever mosquito densities were lower than average. Mosquitoes were identified according to morphological identification keys [55]. Siblings of the *An. gambiae* complex were subsequently identified to species and molecular form by rDNA diagnostic assays [15]. Spatial patterns of occurrence of the *An. gambiae* molecular forms were investigated by spatial autocorrelation analysis. Following procedures described in [56], Moran’s *I* tests of autocorrelation and spatial correlograms were calculated using functions available in the *spdep* and *pgirmess* packages of the *R* software for statistical computing (http://www.R-project.org).

### Land use Classification to Assess Degree of Urbanisation

A satellite image encompassing the study area, taken by Landsat 7 boarding the Enhanced Thematic Mapper Plus (ETM+) observing instrument, was obtained from the University of Maryland’s Global Land Cover Facility (http://www.landcover.org). The scene (path 185 row 57, 30×30 m resolution) was taken on May 18, 2000 (10% cloud cover). This image offered the best compromise between quality and age at the time of analysis as more recent ETM+ images were released only in late 2009 because of malfunction experienced by Landsat 7 since May 2003. We were interested in discriminating the built environment, which includes human infrastructures such as buildings and roads, against natural features, including herbaceous and ligneous vegetation, and water. A “Built Environment Index” (BEI), defined as the proportion of surface occupied by the built environment within a spatial unit, was calculated by extracting the built environment land class from the image.

A subset of the Landsat ETM+ scene matching the study area was processed to generate land use information with the software ENVI v.4.0 (ITT Visual Information Solutions; http://www.ittvis.com). To visualise built-up features, spectral bands 3 (Red), 4 (NIR), and 2 (Green) were mapped in that order to the RGB channels to produce a colour composite in which the built environment and ‘open spaces’ appear in shades of magenta against shades of green characterising natural features.

Thirty polygons (regions of interest), for which *a priori* knowledge about land use was available based on the pixel tonal and textural properties, expert interpretation, and multi-source validation, were used as a training set in a maximum likelihood supervised classification, whose aim is to select pixels in the image having a similar spectral signature. The resulting built environment binary map was validated on the ground at 20 sites, half of which were assigned to the built environment class by the supervised classification. The resulting accuracy of classification was assessed by KAPPA analysis, a discrete multivariate technique used in assessment of agreement, using the *KHAT* statistic, a measure of concordance between the classified and reference data sets [57]. The degree of classification accuracy was strong and significantly better than chance agreement: *KHAT*±SD  = 0.90±0.0970 (*Z* = 9.28; *P*<0.001), a result that is in part due also to the limited number of defined land-use classes (only two: built *vs*. non-built environment).

The layer containing the two land-use classes was overlaid on the sampling map, rendered in vector format using the Spatial Analyst module in ArcGIS, and the surface of the built environment contained within the neighbourhood of each sampled location was calculated to assess the BEI. Neighbourhoods were defined by Voronoi tessellation of the sampling frame containing all the sampled locations. Voronoi tessellation served the purpose of partitioning the study area in ‘zones of influence’ pertaining to each sampled location without resorting to a grid that is arbitrarily positioned over the study area. This was possible because of the regular distribution of the sampled locations. The disadvantage of this approach is that the surface pertaining to each sampling unit is not constant, or–for the few sampled locations defining the convex hull of the study area (in our case, 12 out of 100)–is still somewhat arbitrary due to the positioning of the sampling frame. However, in the micro-geographic and model validation studies, where sampled locations were disposed along a transect or irregularly over the study area (and therefore Voronoi tessellation could not be used), the spatial units used to calculate the BEI were defined by an overlaid grid (cf. further details below).

The procedures described above were applied to calculate the BEI for the meso-geographic survey in the region around Yaounde. The same general procedures were implemented also in the case of the micro-geographic rural *vs.* urban transect, and in the statistical models validation study. A few methodological differences specific to these analyses are described as follows. For the micro-geographic rural *vs.* urban transect, we used a subset of a SPOT-5 (2.5×2.5 m resolution) panchromatic image taken on February 19, 2004, covering a 20×8 km area including the micro-geographic transect. The built environment was extracted by progressively fixing threshold radiometric values (radiometric mask) until a satisfactory map of built *vs*. non-built environment was generated. Each binary map created with a given mask was compared to ancillary data, and the process was iterated until a satisfactory map was obtained. Validation was performed on 50 regions of interest compared with ancillary data. The retained map, obtained applying a radiometric mask ranging 0–150, gave *KHAT* = 0.76±0.064 classification accuracy. In order to compare the degree of concordance between the built environment maps obtained from the ETM+ and SPOT-5 classifications, the two layers were rendered in vector format and overlaid in ArcGIS; the degree of agreement was assessed from the error matrix, which returned 96.1% overall agreement. The spatial units over which the BEI was calculated were 1×1 km quadrats with the sampled locality in the centre. For the statistical models validation study, we used two additional Landsat 7 ETM+ scenes covering the study area (path 185 row 58, and path 186 row 58; Fig. 1A) acquired on 18 March and 26 April 2001 (10%–20% cloud cover). To calculate the BEI, a grid of 5×5 km cells was overlaid to define the ‘zone of influence’ around each point feature identifying a sampled locality.

### Modelling the Probability of Occurrence of the Molecular Forms

The functional relationship defining the probability of occurrence of each form in relation to the Built Environment Index was assessed by binary logistic regression in *R*. Ordinary logistic regression does not take into account spatial dependencies between sites, inducing inflation and spatial autocorrelation of regression residuals. To obviate some of these limitations, we fitted autologistic regression models using a pseudo-likelihood method [58], which adds a spatial dependence term (*autocov*) in the regression equation relating the probability *p* of each form occurrence and the explanatory variables, i.e. log[*p*/(1–*p*)] = *a*+*b*×BEI+*c*×*autocov*, where *a*, *b*, and *c* are regression coefficients, and *autocov* is defined as a weighted average of the number of occupied neighbouring sites amongst a set of *k* neighbours around the focal site: *autocov* = ∑*wy*/*y*, with *y* representing an integer assuming the value of either 0 or 1. The neighbouring sites were defined as those linked to the focal locality according to Delaunay triangulation, and weights *w* were calculated as the inverse of the distance between the focal and each neighbouring site.

### Micro-geographic Rural to Urban Transect

A systematic search for *An. gambiae* indoor-resting adults and larvae in nearby breeding sites was carried out during one week monthly, from May 2008 to April 2009 along a transect running from the central area of Yaounde to the nearest westerly rural villages (Fig. 1E). Yaounde is a town of over 2 million inhabitants situated between 700 and 750 m above sea level. The city is drained by several permanent streams and is situated within the Congo-Guinean phytogeographic zone characterised by a typical equatorial climate with two rainy seasons extending from March to June and from September to November.

Adult mosquitoes were collected by spraying an insecticide aerosol inside the sleeping rooms of 1,481 households (mean±SD per site: 8.5±4.7). Larvae were collected by dipping up to 15 times per breeding site from 3,421 aquatic habitats (mean±SD per site: 285±94, of which 59±23 returned *An. gambiae* larvae). Larval sampling effort was kept as much consistent as feasible across the monthly surveys and sites along the transect: the same team of four collectors inspected all potentially suitable water bodies during approx. 1 hour in each locality. No attempt was made to improve the standardisation of sampling effort and dipping procedures any further, because the only response variables extracted from this data set that were subsequently submitted to statistical analysis were the occurrence or the relative proportion of the two molecular forms, not their abundance. In the absence of prior detailed information regarding the nature of M and S larval habitats in the rainforest of Central Africa, we reasoned it was important to maximise the sample size and the nature of the sampled breeding sites. Thus, only water bodies having a high probability of hosting mosquito larvae were surveyed to optimize the sampling effort deployed. In our experience, it is sound to consider that temporary aquatic habitats have not a high probability to contain mosquito larvae or any other aquatic macro-fauna shortly after their formation. Accordingly, we considered as water bodies potentially suitable to host mosquito larvae those where a conspicuous aquatic macro-fauna was observed. This non-random sampling strategy might have introduced some bias, but its consistent application across all localities should have projected the same bias onto all the sites along the transect. Moreover, the observation that the larval M *vs*. S composition across localities matched almost perfectly that of the independently-collected adult samples (cf. Fig. 1F and S2), indicates that if any bias was introduced, it did not unduly affect the results of the larval surveys.

Larval habitats mostly included rain puddles on bare soil, market-gardening furrows filled with rain and irrigation water, ditches, and waste waters from slums or on dumping grounds. In the rural area, prevailing breeding sites included swamps covered by dense vegetation, and puddles or ditches filled with rainwater. The peri-urban area was characterised by the practice of market-gardening, and the colonisation by the expanding human population of seasonally-flooded lowlands for house construction. Mosquito breeding sites were mostly constituted here by natural sources of water in lowland areas, ditches, and furrows in market-gardening areas. In the urban area, productive mosquito larval habitats were mostly constituted by stagnant water bodies around houses in densely populated lowlands areas, or derived from human activities such as e.g. car washing, and artificial habitats such as e.g. small containers, hollow bricks, abandoned households, or tyres. A more detailed description of the larval habitats included in this study and their properties will be the object of a separate publication (B. Tene-Fossog, C. Antonio-Nkondjio, P. Bousses, N.J. Besansky, C. Costantini, unpublished data), and further details on *An. gambiae* urban larval habitats in Yaounde are available in [45]. Mosquito larvae were morphologically identified, and those belonging to the *An. gambiae* complex were submitted to molecular analysis to establish their species/molecular form status as described previously.

The relative frequency of the two molecular forms pooled across months was plotted for each collection site (Fig. 1F for adults and Fig. S2 for immatures). Whenever a sample returned no individual of one of the two forms (i.e. relative frequency of 0%), the proportion *p* of a population compatible with a sample of size *n* returning no individuals at 99% probability was calculated according to the equation 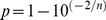 with *n*>0. In other words, there is only a 1% chance of not observing any individual of the focal form in a sample of *n* individuals when the true frequency of the form in the population is *p* [59].

The probability of occurrence of M and S adults along the rural to urban transect was modelled by binary logistic regression. In addition to the BEI, three explanatory variables were fitted to the data: the average mosquito density in the region, expressed as the mean number of *An. gambiae* M or S females per sprayed house (shown in Fig. 2A), the sampling effort deployed in each surveyed site, expressed as the total number of sprayed houses, and an indicator variable assuming the value of one or zero according to whether sites contiguous to the focal one were occupied or not, respectively, by each form. The last variable was meant to account for spatial dependence in occurrence along the linear transect. All explanatory variables, except the spatial dependence indicator variable, explained a significant portion of the total variance (Table S1), and were therefore retained in the final minimal adequate models for both M and S (Table S2).

Following theory on clinal spatial changes in allele frequencies [60], we estimated the width of the M/S hybrid zone, *w*, as the inverse of the maximum gradient of the cline, l/(*dp*/*dx*), where *p* represents the relative frequency of one of the two forms at distance *x* along the transect based on larval samples. The shape of the cline was assumed to follow a logistic curve, and locations along the transect to be separated by approx. 1 km.

### Species Association Analysis

Four surveys in each of the two major urban centres of Cameroon, Douala and Yaounde, were carried out between October 2009 and September 2010 to investigate the nature of larval habitats and the degree of co-occurrence between *An. gambiae* and Culicine mosquitoes that usually breed in habitats highly polluted with organic matter. Overall, 987 water collections localised within the urban city limits were sampled by dipping, and the presence/absence of *An. gambiae* and *Culex* larvae was recorded. Larval habitats included puddles on open grounds or inundated grass-fields embedded within urban infrastructures (65%), ditches (16%), cultivation furrows (8%), ruts (7%), irrigation pits (6%), potholes (2%), and the exposed foundations of houses that were either under-construction, damaged, or abandoned (2%). Sub-samples of *An. gambiae* (total number of identified larvae: *n* = 811) were then molecularly identified to confirm the almost exclusive presence of the M form in both cities. Indeed, S accounted for only 0.5% of these samples.

The binary outcome of joint occurrences from a single survey was organised in a 2×2 contingency table, whose frequencies served to calculate the Hurlbert coefficient of inter-specific association (C_8_). This index possesses a set of desirable properties [33]: it ranges from –1 to +1 (minimal and maximal association under given marginal frequencies), it equals zero when there is statistical independence of occurrence (that is, the frequency of joint occurrences equals that expected from chance alone), and the value is independent of the species (marginal) frequencies. Statistical independence was tested by Fisher’s exact tests.

To relate the degree of species association with the general availability of temporary breeding sites suitable to *An. gambiae*, we calculated an index of aridity according to De Martonne: *DMi* = *P*/(*T*+10), where *P* is the mean monthly rainfall in mm and *T* the average monthly temperature in C corresponding to the time of the year when each of the eight larval surveys were carried out. Rainfall and temperature data were obtained by inverse distance weighting interpolation from the local monthly climate estimator *LocClim* (www.fao.org). It is expected that the greater the aridity–corresponding to periods in the year with less rain and greater evaporation due to higher average temperatures–the lower the availability of temporary larval habitats like e.g. puddles, ruts, or potholes.

## Supporting Information

Figure S1 Spatial autocorrelation in *Anopheles gambiae* molecular forms frequency across the 50×50 km area around the capital of Cameroon, Yaounde. Correlograms of Moran’s *I* index for (**A**) the M form; and (**B**) the S form, with distance classes expressed in meters. Significant autocorrelation coefficients (*P*<0.05) are indicated by closed symbols.
(TIFF)

Figure S2 Spatial distribution of larvae of *Anopheles gambiae* molecular forms along the 16 sites of the micro-geographic rural to urban transect of Fig. 1E. Relative proportion (±95% confidence limits) of M (blue bars) and S (red bars). For those sites giving no measurable frequency of the alternative form, the error bars with asterisks denote the limits of the largest proportion of a population compatible with a sample returning no individuals at 99% probability.
(TIFF)

Figure S3 Receiver Operating Characteristic (ROC) curves of M (blue line) and S (red line) assessing the binary logistic regression models’ predictive accuracy of each form occurrence when applied to the independent data set of surveyed locations from southern Cameroon shown in Fig. 1A. The 45° solid line refers to the null model predicting occurrences at random.
(TIFF)

Table S1 Analysis of deviance of the binary logistic regression models shown in Fig. 3.
(PDF)

Table S2 Regression parameters of the binary logistic regression models shown in Fig. 3.
(PDF)

Text S1 Supplementary results and discussion of the form-habitat relationship based on the Built Environment Index.
(PDF)

Text S2 Methodological limitations of the study.
(PDF)
