# Supplementary figures and images for: Anthropogenic Habitat Disturbance and Ecological Divergence between Incipient Species of the Malaria Mosquito Anopheles gambiae
Source: PLoS One. 2012 Jun 22;7(6):e39453. doi: 10.1371/journal.pone.0039453 (PMC3382172; doi:10.1371/journal.pone.0039453)

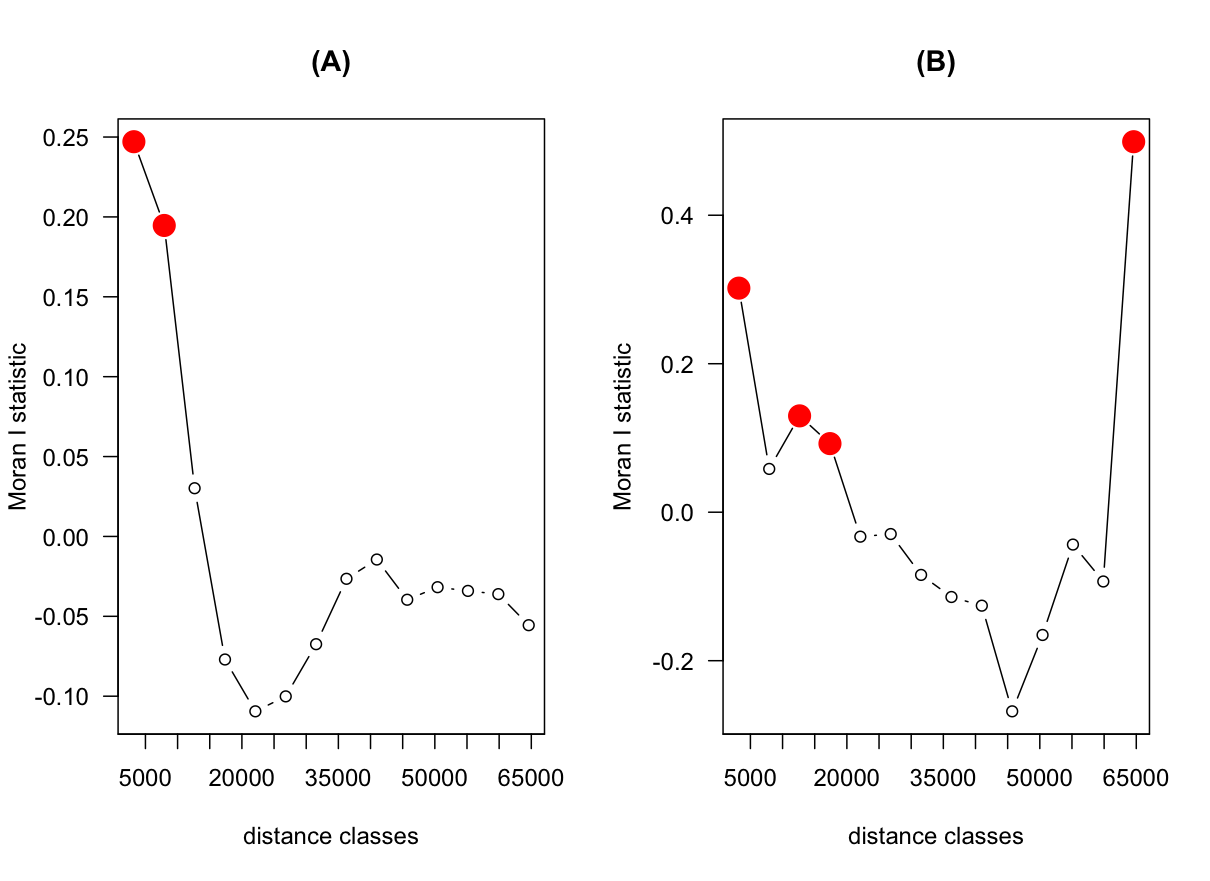

Supplement: Figure S1 — Spatial autocorrelation in Anopheles gambiae molecular forms frequency across the 50×50 km area around the capital of Cameroon, Yaounde. Correlograms of Moran’s I index for (A) the M form; and (B) the S form, with distance classes expressed in meters. Significant autocorrelation coefficients (P<0.05) are indicated by closed symbols. (TIFF) [file pone.0039453.s001.tiff]

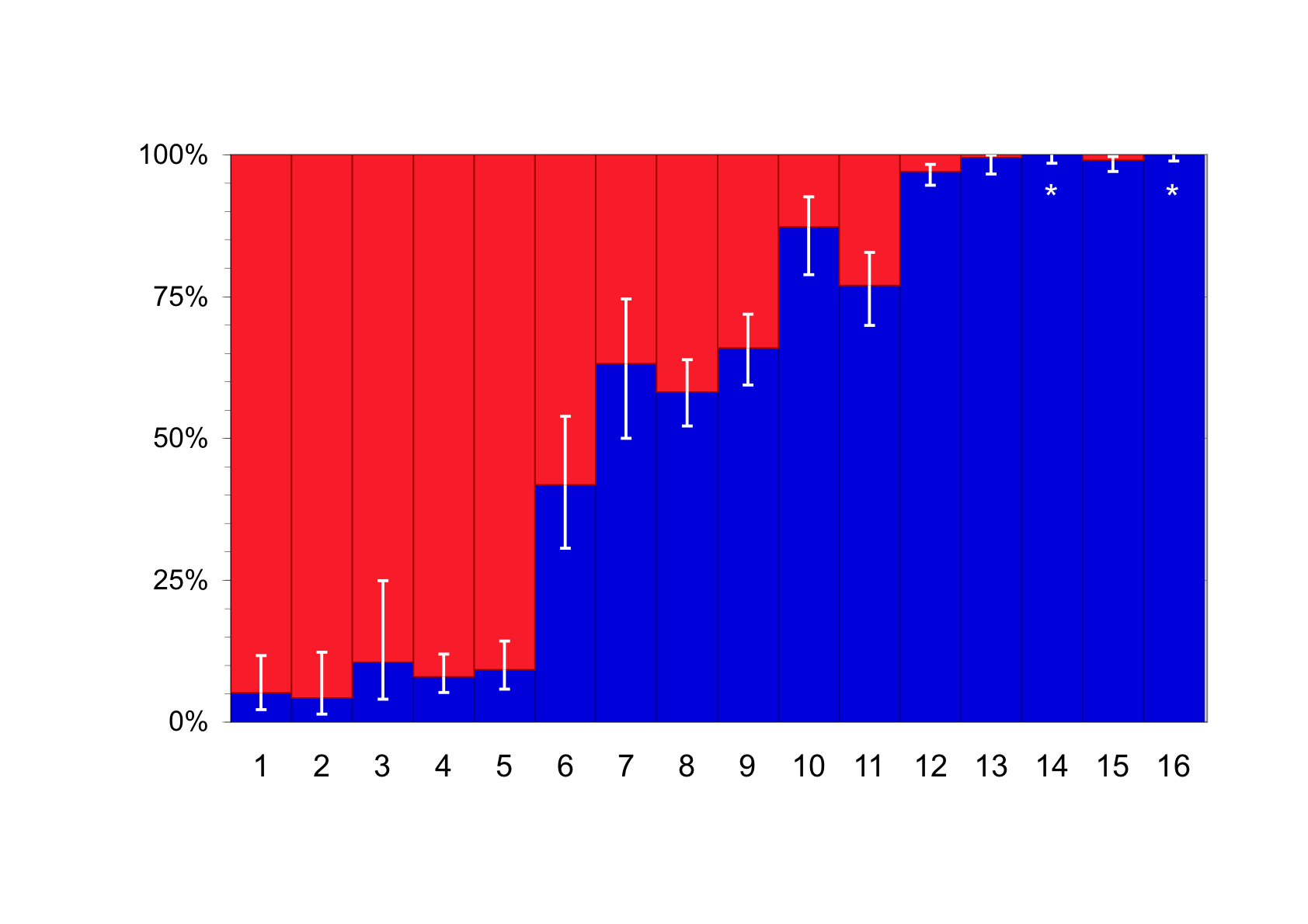

Supplement: Figure S2 — Spatial distribution of larvae of Anopheles gambiae molecular forms along the 16 sites of the micro-geographic rural to urban transect of Fig. 1E. Relative proportion (±95% confidence limits) of M (blue bars) and S (red bars). For those sites giving no measurable frequency of the alternative form, the error bars with asterisks denote the limits of the largest proportion of a population compatible with a sample returning no individuals at 99% probability. (TIFF) [file pone.0039453.s002.tiff]

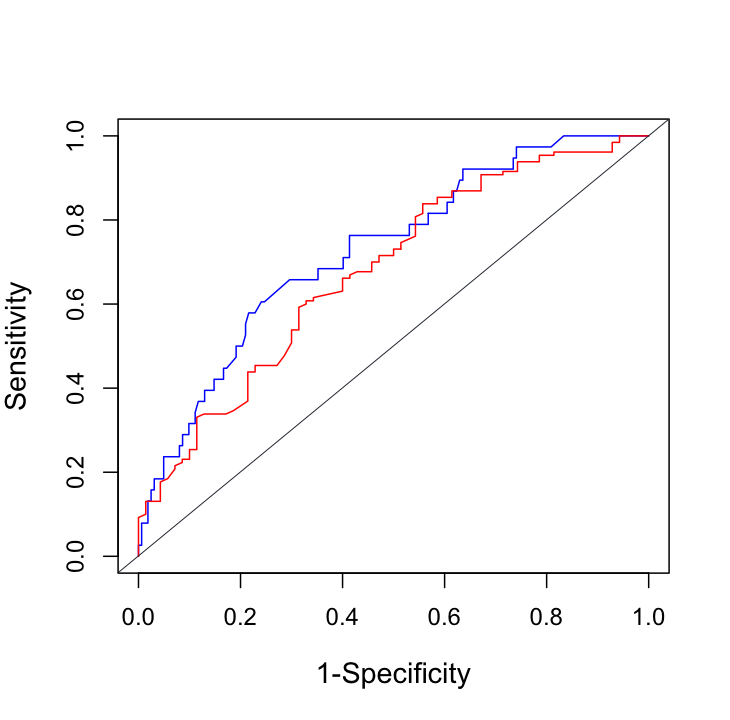

Supplement: Figure S3 — Receiver Operating Characteristic (ROC) curves of M (blue line) and S (red line) assessing the binary logistic regression models’ predictive accuracy of each form occurrence when applied to the independent data set of surveyed locations from southern Cameroon shown in Fig. 1A. The 45° solid line refers to the null model predicting occurrences at random. (TIFF) [file pone.0039453.s003.tiff]
