# Supplementary material for: Anthropogenic Habitat Disturbance and Ecological Divergence between Incipient Species of the Malaria Mosquito Anopheles gambiae
Source: PLoS One. 2012 Jun 22;7(6):e39453. doi: 10.1371/journal.pone.0039453 (PMC3382172; doi:10.1371/journal.pone.0039453)
Supplement: Table S1 — Analysis of deviance of the binary logistic regression models shown in Fig. 3. (PDF) [file pone.0039453.s004.pdf]

| Variable | Form M | | | Form S | | |
|---|---|---|---|---|---|---|
| | Deviance | d.f | *P* | Deviance | d.f | *P* |
| Built Environment Index | 16.81 | 1 | <0.001 | 63.07 | 1 | <0.001 |
| Average Density | 22.92 | 1 | <0.001 | 19.72 | 1 | <0.001 |
| Sampling Effort | 12.83 | 1 | <0.001 | 10.74 | 1 | <0.001 |
| Spatial Correlation | 1.36 | 1 | 0.261 | 0.3 | 1 | 0.515 |
| Residual Error | 199.98 | 187 | | 133.93 | 187 | |
| Total | 256 | 191 | | 244.42 | 191 | |
