# Supplementary material for: Anthropogenic Habitat Disturbance and Ecological Divergence between Incipient Species of the Malaria Mosquito Anopheles gambiae
Source: PLoS One. 2012 Jun 22;7(6):e39453. doi: 10.1371/journal.pone.0039453 (PMC3382172; doi:10.1371/journal.pone.0039453)
Supplement: Table S2 — Regression parameters of the binary logistic regression models shown in Fig. 3. (PDF) [file pone.0039453.s005.pdf]

**Variable**	**Form M**			**Form S**		
	Estimate	SE	*P*	Estimate	SE	*P*
Intercept	-4.184	0.66	<0.001	-1.507	0.522	<0.01
Built Environment Index	1.778	0.47	<0.001	-6.418	1.051	<0.001
Average Density	8.263	1.876	<0.001	5.703	1.391	<0.001
Sampling Effort	0.157	0.045	<0.001	0.187	0.059	<0.01
