# Supplementary material for: Anthropogenic Habitat Disturbance and Ecological Divergence between Incipient Species of the Malaria Mosquito Anopheles gambiae
Source: PLoS One. 2012 Jun 22;7(6):e39453. doi: 10.1371/journal.pone.0039453 (PMC3382172; doi:10.1371/journal.pone.0039453)
Supplement: Text S1 — Supplementary results and discussion of the form-habitat relationship based on the Built Environment Index. (PDF) [file pone.0039453.s006.pdf]

SUPPORTING INFORMATION

Anthropogenic Habitat Disturbance and Ecological Divergence between Incipient Species of the Malaria Mosquito *Anopheles gambiae*

Colince Kamdem, Billy Tene Fossog, Frédéric Simard, Joachim Etouna, Cyrille Ndo, Pierre Kengne, Philippe Boussès, François-Xavier Etoa, Parfait Awono-Ambene, Didier Fontenille, Christophe Antonio-Nkondjio, Nora J Besansky, Carlo Costantini

Text S1

Evaluation of the predictive performance of *Anopheles gambiae* molecular forms distribution models based on the Built Environment Index

The predictive performance of the species distribution models parameterized (Table S2) using M and S occurrences from the micro-geographic survey was evaluated by two approaches: (i) a test of the discriminatory ability of the model to distinguish between occupied and unoccupied sites; and (ii) a test of agreement between observations and predictions ('goodness-of-fit' or model calibration [1]). The validation data-set consisted of independent collections carried out in 200 villages subdivided in two regions lying in the forested domain of southern Cameroon (Fig. 1A). The 100 villages of the first region lie along a transect between LAT 2°20'N –4°30'N and LONG 9°49'E–11°38'E. These locations were surveyed for the purposes of a previous study [2]. Another 100 villages lying between LAT 3°25'N–4°51'N and LONG 10°59'E–11°40'E were surveyed in October-December 2006 following similar sampling procedures. With the exception of four towns (i.e. Bafia, Ebolowa, Kribi, and Mbalmayo—approximate population estimates ranging ~40,000 to ~80,000 people), the sampled areas lack major urban centres (defined as those whose size is >100,000 inhabitants), and therefore their median BEI was quite low (0.012; interquartile range: 0.003-0.037). This is also reflected in the frequency distribution of the predicted probabilities of occurrence of M and S in these areas, which is strongly skewed towards low values for M (in fact, never exceeding 0.5 in the case of this form) and towards high values for S (Fig. S4). Such distributions reflect the general structure of the degree of urbanisation in the forest of Cameroon but, unfortunately, they restrict the refinement of the model evaluation analysis. To our knowledge, however, no other extensive large-scale and accurately georeferenced data-set is available to date for M and S in the forest domain of central Africa that could be used in place.

We assessed how well the model relating the probability of M or S occurrence with the BEI can discriminate between positive and negative sites by the area under the receiver operating characteristic (ROC) curve (AUC–Fig. S3), calculated using the *ROCR* library [3] in *R*. Approximate standard errors (SE) for the AUC were estimated by a non-parametric approach [4]. When the 95% confidence interval of the AUC does not overlap 0.5, it can be assumed that the model can discriminate between negative and positive sites with probability θ=AUC.

To determine whether the addition of the BEI provided a significant improvement in discrimination capacity over a model containing only sampling effort and regional average density of M and S as predictors, the significance of this improvement was calculated using a critical ratio test Z, modified to control for correlation between the two AUCs (with or without the BEI included in the model) calculated using the same validation data-set:

$$Z = \frac{AUC_1 - AUC_2}{\sqrt{SE^2_{AUC_1} + SE^2_{AUC_2} - 2rSE_{AUC_1}SE_{AUC_2}}}$$

The correlation r between the two AUCs was estimated by the mean of the two Spearman rank correlation coefficients calculated between the predictions for occupied sites for each model and the predictions for unoccupied sites for each model. The index was then tested against the normal probability density function to assess statistical significance.

Model calibration was implemented to gauge the contribution of different sources of error in prediction mismatches. A well calibrated model should correctly predict the actual proportion of sites occupied by a focal taxon. Model reliability can be tested by regressing the proportion of occupied sites over predicted probabilities of occurrence, both on the *logit* scale. If the model correctly predicts actual occurrences, the regression line should pass through the origin and have a slope of one. Systematic departures from agreement between observations and predictions are due to bias and spread. The former represents the consistent tendency for the model to under- or over-estimate the proportion of occupied sites, and it is identified by the intercept of the regression line being different from zero. Spread describes departure of the regression line relating observations with predictions from a gradient of 45°. The statistical significance of bias and spread was assessed by likelihood ratio (*LR*) tests [1]. The *LR* tests compare the difference of two deviances to a chi-square distribution. The first test examines the null hypothesis that both the intercept of the regression line is zero and the slope is one (no bias and no spread). The two deviances are that resulting from a regression model forcing the line to pass through the origin and having a slope of one, and that estimating these parameters from the data. This relationship can be written as $D(0,1) - D(a,b)$, with a and b representing the intercept and slope, respectively, of the logistic regression line. The second *LR* test examines the null hypothesis that the intercept is different from zero (i.e. that there is significant bias) given appropriate spread. It is implemented by subtracting the deviance resulting from a model forcing the regression line to have a slope of one to that with both intercept and slope forced to have values of zero and one, respectively: $D(0,1) - D(a,1)$. The last *LR* test examines the null hypothesis that the slope is different from unity (i.e. that there is significant spread) with no bias, and it is calculated as $D(a,1) - D(a,b)$.

The binary logistic models slightly but consistently underestimated the occurrence of the M form (Fig. S5A), and indeed the likelihood ratio tests indicate that there was significant bias but no spread in model predictions (Table S3). Conversely, the model over-estimated the occurrence of the S form at probabilities higher than ~0.15, and possibly underestimated it at probabilities lower than this threshold (Fig. S5B). Both bias and spread were statistically significant, but the impact of bias was well more than that of spread (Table S3).

Calibration bias suggests that the prevalence of the two molecular forms differed between the area where the models were parameterized and those where predictions were applied to. This can arise because of differences in methodology between surveys or between regions of the model

development and evaluation data-sets [1]. Some methodological differences were indeed present between the training and evaluation data-sets: the spatial extent and resolution of the two areas, the resolution of satellite imagery and surface units used to calculate the BEI, and the temporal window for sampling. The two regions were also quite different in their average density of built environment, which impacted the degree of model refinement, as previously discussed.

Moreover, some important predictors are likely to be lacking from the two models. For example, some locations in the evaluation data-set could have different degrees of suitability or be totally unsuitable to *Anopheles gambiae*, which would explain why the model over-estimated occurrences in the case of the S form. The addition of a habitat suitability variable constructed with other eco-geographical descriptors might have improved model predictions, but in this case we *did* want to test for the impact of the BEI alone on model predictions. Similarly, underestimation of M occurrences might have resulted from the habit of this form to expand in less suitable habitat whenever environmental conditions are more favourable, and/or in response to accrued dispersal.

Cited references

1. Pearce J, Ferrier S (2000) Evaluating the predictive performance of habitat models developed using logistic regression. Ecol Model 133: 225-245.
2. Simard F, Ayala D, Kamdem G, Pombi M, Etouna J, et al. (2009) Ecological niche partitioning between Anopheles gambiae molecular forms in Cameroon: the ecological side of speciation. BMC Ecol 9: 17.
3. Sing T, Sander O, Beerenwinkel N, Lengauer T (2005) ROCR: visualizing classifier performance in R. Bioinformatics 21: 3940-3941.
4. Hanley JA, McNeil BJ (1982) The meaning and use of the area under a receiver operating characteristic (ROC) curve. Radiology 143: 29-36.

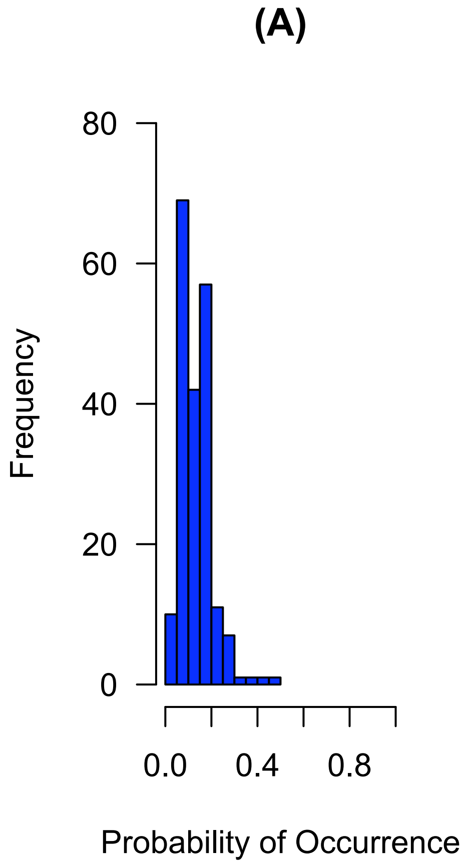

Figure S4. Frequency distribution of predicted probabilities of occurrence of (A) M (blue bars); and (B) S (red bars) according to the binary logistic regression models when applied to the independent data-set of surveyed locations from southern Cameroon shown in Fig. 1A.

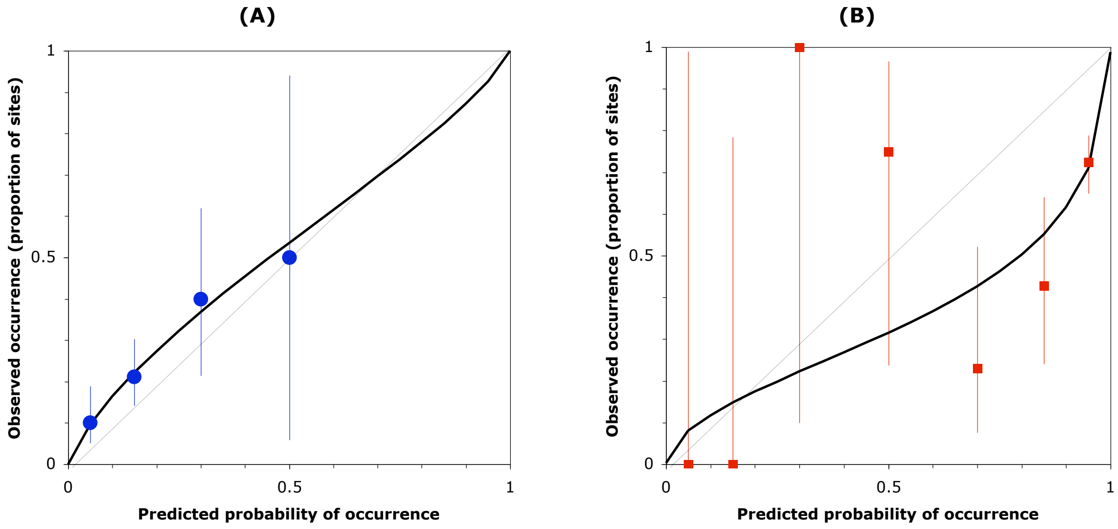

Figure S5. Model calibration analysis of *Anopheles gambiae* molecular form M (A) and S (B). Error bars are 95% confidence limits of the proportion of sites that were found occupied by each taxon for each of the seven bin classes of predicted probability of occurrence. In the case of the M form, no location had a predicted probability of occurrence >0.5, which explains why in this case there are only four classes in the graph. The dotted line at 45° represents perfect agreement between observations and predictions. When the fitted logistic regression line (here represented as a curve on the linear scale) lies above the 45° dotted line, the model underestimates actual occurrences. Conversely, when the regression curve is below the 45° line, the model over-estimates actual occurrences.

Table S3. Statistical inference of model calibration parameters. The first test assumes no bias and no spread as the null hypothesis. The second test examines the null hypothesis of significant bias with appropriate spread. The third test assesses the null hypothesis of no bias and inappropriate spread.

**Test**	**Form M**			**Form S**		
	Deviance	d.f.	*P*	Deviance	d.f.	*P*
**1.** $D(0,1) - D(a,b)$	6.87	2	0.032	110.44	2	<0.001
**2.** $D(0,1) - D(a,1)$	6.33	1	0.012	104.69	1	<0.001
**3.** $D(a,1) - D(a,b)$	0.53	1	0.466	5.76	1	0.016
