# Supplementary material for: Anthropogenic Habitat Disturbance and Ecological Divergence between Incipient Species of the Malaria Mosquito Anopheles gambiae
Source: PLoS One. 2012 Jun 22;7(6):e39453. doi: 10.1371/journal.pone.0039453 (PMC3382172; doi:10.1371/journal.pone.0039453)
Supplement: Text S2 — Methodological limitations of the study. (PDF) [file pone.0039453.s007.pdf]

SUPPORTING INFORMATION

Anthropogenic Habitat Disturbance and Ecological Divergence between Incipient Species of the Malaria Mosquito *Anopheles gambiae*

Colince Kamdem, Billy Tene Fossog, Frédéric Simard, Joachim Etouna, Cyrille Ndo, Pierre Kengne, Philippe Boussès, François-Xavier Etoa, Parfait Awono-Ambene, Didier Fontenille, Christophe Antonio-Nkondjio, Nora J Besansky, Carlo Costantini

Text S1

Methodological limitations of the study

The interval of 4-7 years spanning the time when the satellite images were taken and the time the entomological field surveys took place could bias our analysis if landscape features were significantly altered during this period owing to rapid deforestation and urbanisation. Studies on the urban dynamics of Yaounde reported that the annual rate of urbanisation decreased from 8.1% between 1973-1988 [1] to 3.8% between 1988-1999 [2]. Accordingly, it can be inferred that the urban growth of Yaounde presumably shifted in recent years from spatial extension to increase in density of the built-up surfaces. Given the relatively coarse resolution of the ETM+ remote sensed data, the BEI in this case is less sensitive to fine-scale changes of the built environment. Under these circumstances it is less likely that the pace of urbanisation has unduly affected the general conclusions of our analyses. Even so, the consequence of rapid urbanisation would be an underestimation of the BEI. Considering the mode of growth of Yaounde, this bias would affect more the peri-urban than the other areas (Sietchiping, R. PhD Thesis, University of Melbourne, 2004). The expected effect is that M and S would segregate along the urbanisation gradient with an even steeper slope than that shown in Fig. 1C and Fig. 3.

The model validation analysis showed that the simple species distribution models (SDM) developed for M and S occurrence in the forest of Cameroon based on a single habitat descriptor, i.e. the BEI, together with variables related to the sampling process, i.e. average density and sampling effort, correctly discriminated between occupied and unoccupied sites ~70% of the time, in a different region from that where the SDMs were parameterized. The BEI had a significant impact on this discriminatory ability in the case of the S form. However, significant bias in model predictions for both M and S (slight and positive for M, and marked and negative for S), and some spread error in the case of S were also apparent (cf. Supporting Information Text S1). The intermediate discriminatory power, which is quite frequent in species distribution modelling [3], and the presence of bias call for further refinement of our SDMs in both ecological predictors and assessment of land cover [4]. Nonetheless, it is promising that a single habitat classifier extracted from medium resolution satellite imagery could discriminate occurrences of M and S in a consistent manner and with sufficient accuracy, even after extrapolation to a different study area and using somewhat different methodologies.

The BEI is a simple index defined on per-pixel values which does not incorporate contextual information. Under certain circumstances, therefore, a set of scattered villages could potentially return the same BEI as one isolated larger town. The outcome depends mostly on the size and shape of the spatial unit of reference over which the BEI is calculated relative to the spatial structure and size of the built-up surfaces that are subtended. In our study, the adopted spatial unit of reference

changed across surveys: the meso-geographic survey used Voronoi tessellation, the micro-geographic transect 1×1 km buffer cells, and the SDM evaluation study a grid of 5×5 km cells (see Methods). Findings from all three approaches provided similar results, demonstrating that general conclusions were robust despite the limitations inherent in the BEI. It is worth noting that the density of built environment represents a descriptor correlated with the proximate causes of M and S ecological divergence. Accordingly, the significant association that was found between M or S occurrence and the BEI remain valid whatever the spatial properties or interpretations that can be attributed to the BEI.

Cited references

1. Sietchiping R (2003) Urban evolution of Yaoundé (Cameroon) between 1973 and 1988 by remote sensing. Télédétection 3: 137-144.
2. Sietchiping R (2001) [Assessing urban dynamics in tropical areas using Landsat images: application to the city of Yaoundé, Cameroon]. IXème Journées Scientifiques du Réseau Télédétection Yaoundé.
3. Segurado P, Araujo M (2004) An evaluation of methods for modelling species distributions. J Biogeogr.
4. Tatem AJ, Hay SI (2004) Measuring urbanization pattern and extent for malaria research: a review of remote sensing approaches. J Urban Hlth 81: 363-376.
